# From Craniotomy to Caesarean: A Multidisciplinary Approach to Meningioma During Pregnancy

**DOI:** 10.7759/cureus.86469

**Published:** 2025-06-21

**Authors:** Mohammedelfateh Adam, Joshil Selva Jothi, Karim Botros, Azriny Khalid

**Affiliations:** 1 Obstetrics and Gynaecology, University Hospital Galway, Galway, IRL; 2 Obstetrics and Gynaecology, Cork University Maternity Hospital, Cork, IRL; 3 Obstetrics and Gynaecology, Poole Hospital, University Hospitals Dorset NHS Foundation Trust, Bournemouth, GBR; 4 Obstetrics and Gynaecology, University Hospital Waterford, Waterford, IRL

**Keywords:** brain tumour, brain tumour surgery, high-risk pregnancy, multidisciplinary team, pregnancy cancer, safe outcome

## Abstract

This case report addresses a brain tumour - specifically, a meningioma - occurring during pregnancy. It describes the initial diagnosis, management, and outcome of a woman in her early 40s. She is para 2, and at 24 weeks + 3 days’ gestation, she presented to the Emergency Department with confusion and neurological deficits. A computed tomography (CT) scan of the brain revealed a 6.4 cm meningioma in the left frontal lobe. Following a multidisciplinary discussion, the obstetric and neurosurgical teams opted for surgical intervention: a bi-frontal craniotomy with Simpson Grade I resection, performed at 25 weeks’ gestation. The post-surgical phase resulted in a positive pregnancy outcome, with the patient delivering a healthy male infant at 38 weeks via planned lower-segment caesarean section. This case highlights the potential for successful obstetric outcomes in pregnant women with brain tumours undergoing second-trimester surgical intervention, potentially negating the need for pregnancy termination.

## Introduction

Meningiomas are the most common type of benign, non-glial brain tumour. They are more common in postmenopausal women and are typically seen between the ages of 40 and 60. Meningiomas are 90% benign, 6% atypical, and 2% malignant [1]. They are derived from arachnoid cap cells and can be diagnosed based on symptoms or incidentally [2]. Intracranial tumours are rare during pregnancy. According to Isla et al. [3], 12 pregnant women were diagnosed with intracranial tumours over 12 years, and only two cases were meningiomas. However, it is believed that there is a strong association between female cancers and hormones [4].

Meningiomas are more common in women and have been shown to grow more rapidly during the luteal phase of the menstrual cycle and during pregnancy [5,6]. Common symptoms include headache, confusion, memory problems, nausea, vomiting, and occasionally visual or speech disturbances. These symptoms are often related to raised intracranial pressure. The growth of the tumour is thought to be due to water retention, increased blood flow, and the presence of sex hormone receptors on the tumour cells. Early signs, such as persistent nausea and vomiting, may be misinterpreted as hyperemesis gravidarum, particularly in the first trimester [7]. It is always difficult for the doctor and the patient to decide whether the pregnancy should be terminated. A case of brain meningioma causing confusion and memory changes during pregnancy is described in this report. This case also addresses the initial diagnosis, management, and pregnancy outcomes.

## Case presentation

The patient is in her early 40s, with a normal body mass index. She is para 2 and has had two previous spontaneous vaginal births at term, both following uncomplicated pregnancies. She has no significant gynaecological history. Her medical history includes asthma, but she has no other significant conditions. She has no history of alcohol use, substance abuse, or smoking. She is otherwise fit and leads a healthy lifestyle.

The routine dating ultrasound findings were normal. There were no obstetric concerns during the booking visit. The anatomy ultrasound scan at 21 weeks was normal. Although there were no obstetric concerns, there were worries about her comprehension of speech over the last few weeks and her forgetfulness in daily tasks. She had no headaches, seizures, palpitations, or visual disturbances. The neurological examination showed good speech comprehension and orientation to time and place. She declined any further medical review or perinatal mental health review. Therefore, she was discharged home and scheduled for follow-up in the antenatal clinic as planned.

At 24 weeks + 3 days of gestation, the patient presented to the Emergency Department, complaining of feeling unwell - generally nonspecific - for two days. She could not respond verbally, appeared confused and disoriented about place and time, and failed to answer any questions clearly. Obstetric examination showed no significant foetal compromise, and ultrasound findings were normal. Given the ongoing confusion, a perinatal mental health review was arranged. During this review, the patient could not finish sentences and was forgetful. She denied any suicidal thoughts, self-harm, or previous psychiatric problems. The perinatal mental health team advised that physical causes must be investigated further to rule out neurological causes, as her condition was unlikely to be due to a primary psychiatric reason.

We arranged a review by the medical team, and they requested an urgent computed tomography (CT) scan of the brain. On examination by the medical team, there was no facial droop; speech or swallow disturbances; limb weakness; sensory disturbances; photophobia; neck stiffness; or vertigo symptoms. The patient correctly answered the day and month but failed to state the year. Pupils were equal and reactive to light and accommodation (PEARLA), and cranial nerves II through XII were intact. Speech was normal, and the patient could follow three-step commands; however, assessment of dysdiadochokinesia was challenging, as she was unable to follow instructions consistently.

Investigations

The patient’s blood work - including complete blood count, urea and electrolytes, liver function tests, and thyroid function tests - was all within normal ranges (Table 1).

**Table 1:** Patient's blood results. ALT, Alanine Aminotransferase; ALP, Alkaline Phosphatase; Gamma GT, Gamma-Glutamyl Transferase; eGFR, Estimated Glomerular Filtration Rate; CRP, C-Reactive Protein; TSH, Thyroid-Stimulating Hormone; Free T4, Free Thyroxine; WBC, White Blood Cells; RBC, Red Blood Cells; Hb, Haemoglobin; HCT, Haematocrit; MCV, Mean Corpuscular Volume; MCH, Mean Corpuscular Haemoglobin; MCHC, Mean Corpuscular Haemoglobin Concentration; APTT, Activated Partial Thromboplastin Time

Test	Result	Units	Normal Range
Urea	3.9	mmol/L	2.50 - 7.80
Sodium	138	mmol/L	135.0 - 145.0
Potassium	4.2	mmol/L	3.50 - 5.30
Chloride	103	mmol/L	95.0 - 108.0
Creatinine	57	µmol/L	45.0 - 84.0
eGFR	>90	mL/min/1.73 m²	>90
ALT	23	U/L	5.0 - 33.0
Total Bilirubin	4.3	µmol/L	2.0 - 21.0
ALP	82	IU/L	30.0 - 130.0
Gamma GT	88	U/L	6.0 - 42.0
Total Protein	77	g/L	60.0 - 80.0
Albumin	45	g/L	35.0 - 50.0
Calcium	2.34	mmol/L	2.20 - 2.60
Phosphorus	1	mmol/L	0.8 - 1.5
Magnesium	0.9	mmol/L	0.7 - 1.0
CRP	1.8	mg/L	0.0 - 5.0
WBC	6.8	x10⁹/L	4.0 - 10.0
RBC	4.83	x10¹²/L	3.8 - 4.80
Hb	13.6	g/dL	12.0 - 15.0
HCT	0.42	L/L	0.36 - 0.46
MCV	86.3	fL	83.0 - 101.0
MCH	28.2	pg	27.0 - 32.0
MCHC	32.6	g/dL	31.5 - 36.0
Platelets	310	x10⁹/L	150 - 400
Neutrophils	3.47	x10⁹/L	2.0 - 7.00
Lymphocytes	2.37	x10⁹/L	1.0 - 3.00
Monocytes	0.52	x10⁹/L	0.20 - 1.00
Eosinophils	0.37	x10⁹/L	0.02 - 0.50
Basophils	0.05	x10⁹/L	0.02 - 0.10
Prothrombin Time	9.4	seconds	8.7 - 12.70
APTT	27	seconds	22.00 - 34.00
TSH	2.1	mIU/L	0.4 - 4.0
Free T4	14.2	pmol/L	9.0 - 25.0

**Figure 1:**
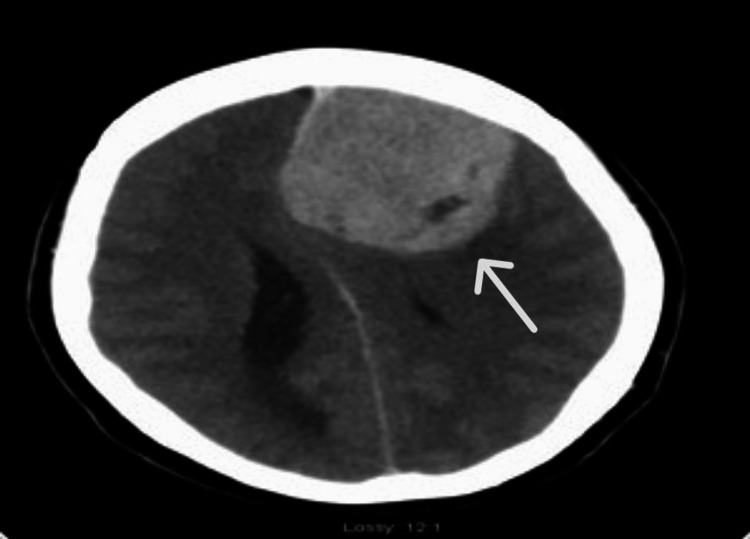
A CT brain illustrates a well-defined enhancing lesion in the left frontal lobe with mass effect and shift of the midline falx (arrow). CT, Computed Tomography

A CT brain imaging, pre- and post-contrast study, showed a 6.4 × 5.4 × 6.1 cm contrast-enhancing mass overlying the left frontal region, crossing the falx cerebri, and causing extensive sulcal effacement and vasogenic oedema. There was also a midline shift of 1.1 cm to the right, with internal foci of calcification and haemorrhage within the lesion, and complete effacement of the anterior horns of both lateral ventricles and the third ventricle. The prominence of the temporal horns and extensive transependymal oedema were consistent with obstructive hydrocephalus. There was uncal herniation, but no cerebellar tonsillar herniation. The CT scan showed regular appearances of the brainstem and cerebellum. The orbits and periorbital structures were normal in appearance. The paranasal sinuses and mastoid air cells were well aerated, and there were no fractures. The impression was a 6.4 cm enhancing mass overlying the left frontal lobe, with involvement of the falx cerebri and adjacent hyperostosis. The features were most in keeping with meningioma (Figure 1). There was obstructive hydrocephalus, midline shift, and uncal herniation. Hence, urgent neurosurgical input was suggested.

**Figure 2:**
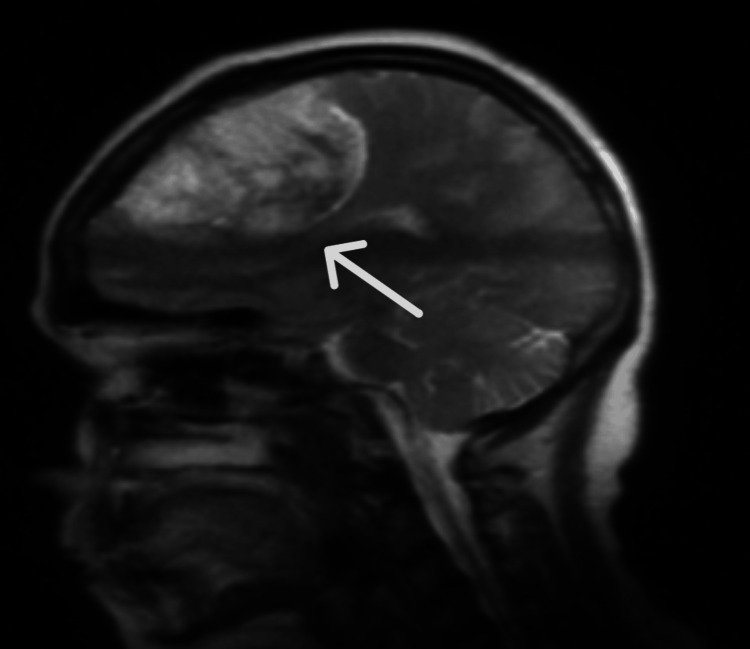
A T2-weighted sagittal MRI image showing a well-defined mass in the frontal region with its base to the periphery of the meninges (arrow). MRI, Magnetic Resonance Imaging

**Figure 3:**
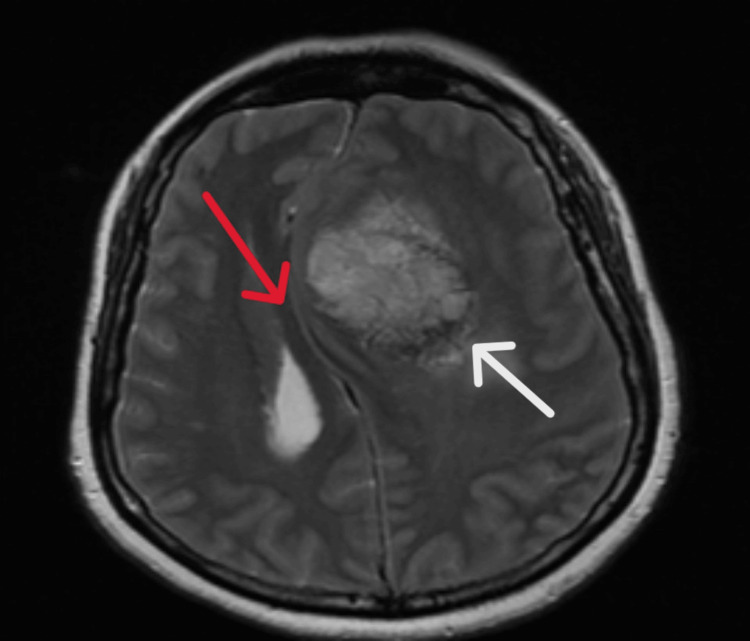
A brain MRI demonstrating a left frontal mass (white arrow), with surrounding oedema and mass effect on the midline dura (red arrow). MRI, Magnetic Resonance Imaging

**Figure 4:**
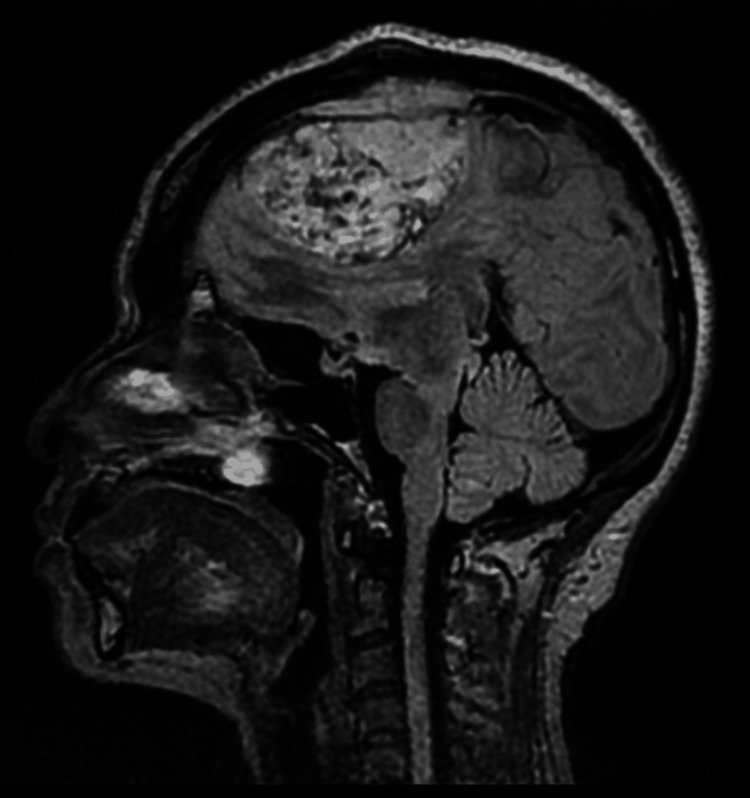
MR venogram of the brain shows a large, extra-axial, left frontal mass lesion. MR, Magnetic Resonance

Following a discussion with the neurosurgical team at the tertiary unit, an urgent transfer for further management was scheduled. Based on their recommendation, we commenced the patient on intravenous dexamethasone 8 mg and anti-epileptics (levetiracetam) 500 mg intravenously as prophylactic doses. Next, before any intervention, we performed a non-contrast magnetic resonance imaging (MRI) of the brain and a venogram, which showed a sizable extracranial mass lesion in the left frontal lobe, parasagittally. Heterogeneous enhancement was seen, likely representing a focal area of haemorrhage. The mass measured approximately 8.0 × 5.0 × 5.0 cm, as shown in Figures 2-4. There was some oedema present in the adjacent left frontal lobe. Overall, the findings indicated a large extra-axial mass, most likely a meningioma, involving the middle portion of the superior sagittal sinus and the parietal bone, resulting in a significant mass effect.

Treatment

**Figure 5:**
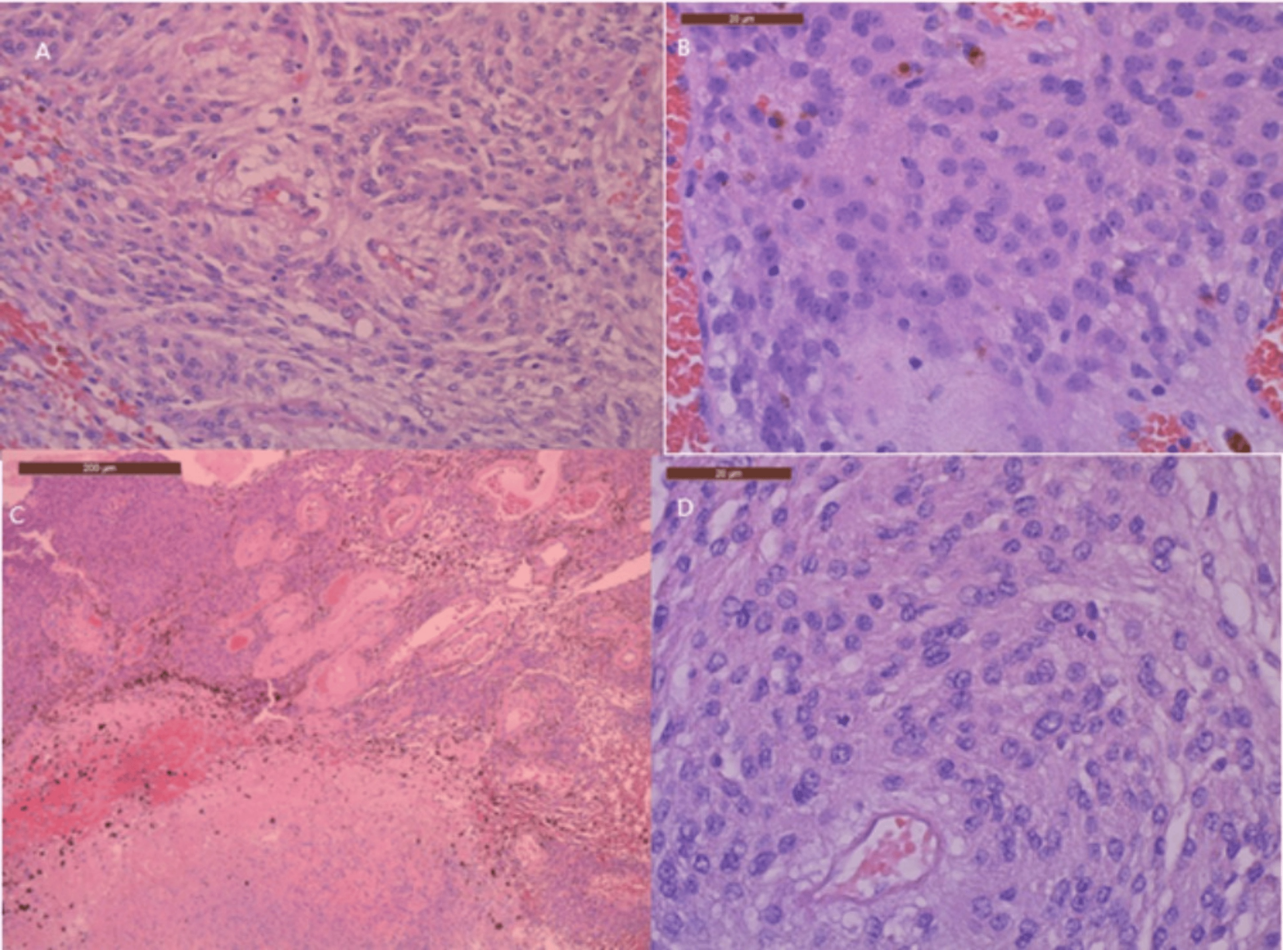
(A) Meningioma showing prominent nucleoli (B), confluent necrosis (C), and mitotic activity (D), corresponding to atypical meningioma, CNS WHO Grade 2. CNS WHO Grade 2, Central Nervous System World Health Organization Grade 2

Given her significant speech and thought process deterioration, the neurological deficit in memory, and the constant state of confusion, an intervention was needed. A multidisciplinary team meeting was scheduled, comprising obstetric, neurosurgical, and anaesthetic teams. They made their decision to proceed with the surgical management option without terminating the pregnancy. A bi-frontal craniotomy with Simpson Grade I resection of the frontal lobe meningioma was performed. The surgery lasted approximately six hours under general anaesthesia. The histology report confirmed a meningioma with atypical morphological findings, consistent with an atypical meningioma (World Health Organization (WHO) Grade 2), as shown in Figure 5. The immunohistopathological examination showed mild nuclear staining and a positive response to progesterone receptors (PRs).

**Figure 6:**
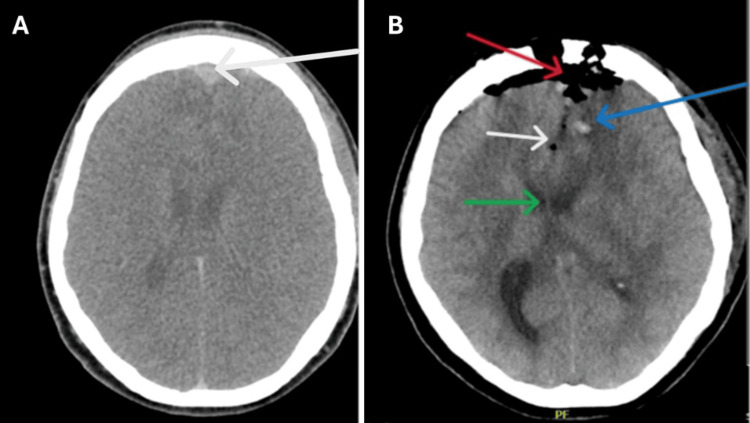
Postoperative CT brain. (A) CT brain shows evidence of a thin subdural density collection over the frontal lobe (white arrow). (B) CT brain image shows a frontal craniotomy with pneumocephalus (red arrow), small-volume haemorrhage (blue arrow), intracranial air (white arrow), and residual midline shift to the right (green arrow). CT, Computed Tomography

During postoperative recovery, from day 1 to day 3, she was doing well, and no concerns were expressed. Ultrasound post-surgery showed an active foetus with an estimated foetal weight (EFW) of 724 g. On day 5 post-operation, no obstetric problems were emerging, but confusion was still present. Further management was discussed with the neurosurgical team; possible confusion was due to cerebral oedema. Brain CT findings noted expected post-surgical changes within the operative bed, with small-volume haemorrhage, intracranial air, thin subdural density collections over the frontal lobes, residual midline deviation to the right side, but no hydrocephalus (Figure 6).

We reduced the steroid medication while continuing the antiepileptic medication (levetiracetam). We managed the patient conservatively and performed a therapeutic lumbar puncture and electroencephalography (EEG), both of which showed normal findings. By day 14 post-surgery, the patient was oriented to time, person, and place, and was aware of the pregnancy. Therefore, we discharged her home and arranged outpatient follow-up with the neurology clinic and the high-risk antenatal clinic.

Outcome and follow-up

Although the patient had significant brain surgery antenatally, she could continue her pregnancy without any adverse outcomes. On follow-up, she had no neurological deficits. She came off steroids but continued taking anti-epileptics and has had no seizures since the operation. The multidisciplinary team recommended that the patient undergo an MRI post-delivery and that her case be re-discussed for adjuvant radiotherapy, if needed.

From her pregnancy outcome point of view, the obstetric team reviewed the patient as routine in the outpatient high-risk antenatal clinic. All antenatal visits had no obstetric concerns, and the patient had a planned elective lower-segment caesarean section at 38 weeks of gestation under a spinal anaesthetic, which was uncomplicated. She requested bilateral tubal ligation to be performed during the caesarean section, as she was not planning to have more children. On day 3 post-caesarean section with bilateral salpingectomy, she was well-oriented and had no neurological deficits. She was discharged home with a follow-up planned with the neurosurgical team for further management.

## Discussion

Meningiomas that arise from arachnoid cap cells are usually found in the skull base and perivenous sinuses, where these cells are abundant [7]. Although most meningioma cases are sporadic, people with the neurofibromatosis-2 gene are at higher risk [8]. However, the role of sex hormones remains unclear. In 1898, Bernard was the first to identify a meningioma during pregnancy [9]. Meningioma enlargement during pregnancy is commonly associated with breast cancer in women and contains oestrogen and PRs. This leads to the conclusion that there could be a correlation between pregnancy and meningioma [4,10,11]. These tumours grow slowly, but pregnancy accelerates this process, causing symptoms. Although radiotherapy is a treatment option for meningiomas, surgery is usually preferred. This decision is based on symptoms, age, radiological findings, postoperative morbidity, patient preference, and the need for a definite diagnosis [7]. The decision regarding surgical timing of meningioma diagnosed during pregnancy also depends on immunohistochemical assessment, particularly for PR receptor positivity [12]. The meningioma in this patient measured 8 x 5 x 5 cm and contained heterogeneous enhancement, likely focal areas of haemorrhage, and significant mass effect.

The patient and the foetus must be evaluated when a meningioma is discovered during pregnancy. A multidisciplinary decision should be made regarding an operation, considering the patient, the foetus, and the family’s decision. Surgical excision without terminating the pregnancy must be handled with caution, considering intraoperative blood loss, hypotension, hypovolemia, and hypoxia. Corticosteroids can be used safely during the postoperative and post-partum periods. A dose of 4 mg dexamethasone every six hours is usually preferred. Mannitol can cross the maternal-foetal barrier; therefore, it should be used only in life-threatening situations. For seizures, monotherapy should be preferred. We should remember that seizure complications are more severe than antiepileptic side effects [13].

When a meningioma is discovered during pregnancy, the surgeon must decide whether to perform surgery immediately or wait until the pregnancy is over. In the event of an emergency, such as widespread oedema, midline shift, change in consciousness, paresis, acute hydrocephalus, or acute total neurological deficit, dual surgery can be performed in which the pregnancy is terminated as the tumour is removed. If the relatives decline termination of the pregnancy, a high-risk tumour excision may be performed while the foetus’s condition is closely monitored. However, the family should be made aware of the potential dangers.

When a benign lesion, such as a meningioma, is discovered during pregnancy and the patient is neurologically stable with no deficit, close follow-up and an MRI would be sufficient to allow the pregnancy to proceed normally. Although there is no decisive impact of surgical management of the meningioma on the mode of delivery [4], a caesarean section can be considered to avoid the Valsalva manoeuvre in the second stage of labour, which could further increase the already elevated intracranial pressure. When evaluating such patients, the primary concern should be maternal health status and the prognosis of the disease. In the event of a life-threatening condition or a severe neurological deficit, follow-up is out of the question, and the lesion must be operated on, where the foetus’s status is not a priority.

## Conclusions

This case illustrates the effective, multidisciplinary management of an atypical meningioma identified during pregnancy. Early imaging, collaborative planning, and timely surgical intervention enabled safe treatment while maintaining the pregnancy. This has led to a positive outcome for both the mother and the baby.
